# A Syllabus of New Remedies and Therapeutic Measures

**Published:** 1901-11

**Authors:** 


A Syllabus of New Remedies and Therapeutic Measures. With chemistry, physical appearance and therapeutic application. By J. W. Wainwright, M. D. Duodecimo, pp. 224. Chicago: G. P. Engelhard & Co. 1901.

The preface of this little work explains itself better than a reviewer's pen can do, and the following abstract will show the aim and scope of the book:

Synthetic chemistry has added many new remedies to the physician's armamentarium, and the knowledge of these becomes day by day more extended as their application is better understood, but the curricula in our schools do not include them, many otherwise admirable books ignore them, and thus force the progressive practitioner to accept interested literature or the statement of, usually, non-professional men. It may be asked from what source can one obtain authoritative and disinterested information regarding these products? There can be but one answer: from the published clinical reports appearing in medical literature, mostly journals, from time to time. Yes! but the busy practitioner or student does not have the opportunity, the time or means to consult this great number of journals or works, nor to condense the information therein contained into a compass sufficiently brief, yet comprehensive to furnish him with the practical or useful part. It has been the aim of the author to do this conscientiously, accurately, and with due regard to the wants of the busy man.

The articles treated of are few, when compared with the multitude which are offered the profession, and only those, therefore, which have passed the experimental stage, which have become necessary ones, are included. Further, only those products whose chemistry is known, or whose exact formula are given, appear. Therapeutic measures other than those coming from the laboratory are also treated of, as a number of them have a most important place in the therapeutics of today.

